# Comparison of the systemic phospholipid profile in dogs diagnosed with idiopathic inflammatory bowel disease or food-responsive diarrhea before and after treatment

**DOI:** 10.1371/journal.pone.0215435

**Published:** 2019-04-16

**Authors:** Katja Kalenyak, Romy M. Heilmann, Chris H. A. van de Lest, Jos F. Brouwers, Iwan A. Burgener

**Affiliations:** 1 Department for Small Animals, Veterinary Teaching Hospital, College of Veterinary Medicine, University of Leipzig, Leipzig, Saxony, Germany; 2 Faculty of Veterinary Medicine, Department of Biochemistry & Cell Biology, Lipidomics Facility, Utrecht University, CM Utrecht, Netherlands; 3 Division of Small Animal Internal Medicine, Department for Companion Animals and Horses, Vetmeduni Vienna, Vienna, Austria; "INSERM", FRANCE

## Abstract

**Background:**

Inflammatory bowel disease (IBD) and food-responsive diarrhea (FRD) are common chronic enteropathies in dogs, of which the exact pathogenesis has not been fully understood. In people dyslipidemia has been reported in patients with IBD, and potential therapeutic benefits of polyunsaturated fatty acids (PUFA) in the treatment of IBD have been investigated. Studies on the phospholipid profile in dogs with IBD and FRD are still lacking.

**Aim:**

To investigate the systemic phospholipid profile of dogs with IBD or FRD and to evaluate possible differences in phospholipids before and after treatment.

**Methods:**

The phospholipids in whole blood and EDTA plasma of 32 dogs diagnosed with either IBD (n = 16) or FRD (n = 16) were analyzed by hydrophilic interaction liquid chromatography (HILIC) prior to and after initiation of treatment, which included an elimination diet enriched with PUFAs.

**Results:**

A clear separation of the phospholipids between whole blood and plasma was demonstrated on principal component analysis plots. In addition to the type of specimen, treatment and disease severity were the most significant factors determining the variance of the phospholipid profile. An increase in lysolipids was observed after treatment. The phosphatidylcholine (PC) species changed from PC 38:4 before treatment to mainly lysophosphatidylcholine 18:0 after treatment. Furthermore, several differences in the abundance of individual phospholipids were identified between dogs with IBD and dogs with FRD and between treatment statuses using random forest analysis.

**Conclusion:**

Significant variances were identified in the phospholipid profiles of dogs with IBD and FRD. These were particularly determined by type of specimen used, disease severity and treatment status. After treatment, a shift of phospholipid species towards lysophosphatidylcholine 18:0 was observed. Future studies should further investigate the role of lipids in the pathophysiology of IBD and FRD as well as their potential therapeutic benefits.

## Introduction

Chronic inflammatory enteropathies (CIE) are a group of common disorders in dogs, which are categorized based on the patient’s response to treatment as either food-responsive diarrhea (FRD), antibiotic-responsive diarrhea (ARD), or idiopathic inflammatory bowel disease (IBD) [1–4]. Dogs with FRD will show a complete clinical response after dietary modification to a novel source of protein and carbohydrates or to a commercially available hydrolyzed protein diet [5,6], whereas dogs with ARD require the use of antibiotic treatment, for example with tylosin, in addition to dietary management for clinical signs of gastrointestinal disease to resolve [7–9]. Idiopathic IBD is defined as chronic gastrointestinal signs of a complex pathogenesis, histologic confirmation of intestinal inflammation, and the necessity for anti-inflammatory and / or immunosuppressive treatment [2,4,7,10]. To date, the etiopathogenesis of CIE, in particular of idiopathic IBD, has not been fully unraveled. However, the current state of knowledge strengthens the notion that a combination of a genetic susceptibility [11–15], dietary and environmental factors, the intestinal microbiota, and an exaggerated immune response contribute to the development of idiopathic IBD in dogs [16–20]. This complexity involving the pathogenesis of IBD urgently asks for potential novel treatment strategies in addition to the currently used stepwise treatment approach of dietary modification, antibiotic trials, and immunosuppressive treatment [2,3]. Novel approaches, including beneficial alterations in the intestinal microbiota through the administration of probiotics and / or prebiotics [21–26] or fecal microbial transplants [27–29], have recently attracted great attention and warrant further research to fully elucidate their therapeutic potential or benefit.

In human medicine, alterations in lipid profiles and lipid homeostasis have been reported with several diseases, including metabolic syndrome [30], diabetes mellitus [30,31], myocardial infarction [31], Alzheimer’s disease [32,33], and cancer [34]. Likewise, dyslipidemia has been detected in patients diagnosed with IBD [35–37]. Similar to the findings in humans, altered lipid profiles have been recognized in dogs diagnosed with idiopathic hyperlipidemia [38,39], diabetes mellitus [40], parvoviral infection [41], cancer [42], renal disease [43,44], or systemic infections [45]. Besides possibly contributing to the pathogenesis of those diseases, several studies have demonstrated lipids, in particular polyunsaturated fatty acids (PUFA), to also have immunomodulatory, anti-inflammatory, and potentially other beneficial effects both in human [46–48] and in veterinary patients [49,50]. These characteristics of PUFAs appear to be very promising from a therapeutic and even a preventative [51] perspective. However, there is currently only very limited data suggesting that dietary supplementation with PUFAs yields a clinical benefit in humans with IBD [36,47,48]. In veterinary medicine, only two studies have investigated the effect of supplemental PUFAs in dogs diagnosed with CIE. Those studies indicate that adding PUFAs to the diet might modify cholesterol homeostasis [52] and also modulate the expression of genes affecting intestinal fatty acid uptake [53], both of which appear to be beneficial in dogs with CIE.

Phospholipids are amphiphilic lipids and represent fundamental components of biological membranes, where they are organized as lipid bilayers [54,55]. Due to their essential abundance in cell membranes their fatty acid composition has a major influence on membrane quality and phospholipids further serve as sources of fatty acids, including PUFAs, lipid mediators and molecules of cell signaling [55,56]. Thus, phospholipids and their membrane composition have great influence on health [55] and, similar to PUFAs, they have been reported to have beneficial effects in several diseases in humans [54,57–61].

To the authors’ knowledge, the systemic phospholipid profiles have not been reported in dogs with CIE (neither before nor after initiation of treatment). Therefore, the objectives of this study were (1) to compare the phospholipid profiles between dogs with idiopathic IBD and dogs diagnosed with FRD, and (2) to evaluate the effect of treatment including dietary supplementation with PUFAs on the phospholipid composition in dogs with CIE by comparing the phospholipid profiles before and after induction therapy. It was hypothesized (1) that the phospholipid profiles differ between the two disease categories, and (2) that the phospholipid profiles also differ within each disease group depending upon the treatment status.

## Materials and methods

### Animals and study protocol

Stored whole blood and plasma samples of a previously reported study on canine chronic enteropathies by one of the authors (Iwan A. Burgener) [11,62] were used for the current investigation. In that original study, dogs with chronic gastrointestinal signs, in the form of diarrhea with or without vomiting or weight loss for at least six weeks, were prospectively enrolled between December 2006 and November 2008. Additional inclusion criteria comprised the absence of an identifiable underlying disorder, histopathologic evidence of intestinal inflammation, and no treatment with antibiotics, corticosteroids, antisecretory medications, or combinations of these for at least two weeks prior to enrollment of dogs into the study. As most dogs had already received dietary modifications prior to referral, previous dietary trials did not preclude a dog’s participation in this project. To exclude possible underlying disorders, a complete blood count, serum biochemistry profile, urinalysis, measurement of serum canine trypsin-like-immunoreactivity (cTLI), serum cobalamin and folate concentrations, adrenocorticotropic hormone stimulation test, parasitic and bacterial fecal examination (including *Clostridium* spp, *Campylobacter* spp and *Salmonella* spp), abdominal ultrasonography, and endoscopy of the gastrointestinal tract were performed in all dogs. The specific canine pancreatic lipase test was not readily available in Europe between 2006 and 2008. Thus, a diagnosis of pancreatitis was ruled out based on a normal serum amylase and lipase activity, a normal serum cTLI concentration, and absence of abdominal ultrasound findings consistent with pancreatitis. All dogs were treated with an antiparasitic (fenbendazole 50 mg/kg p.o. SID for 5 days) irrespective of the results of the fecal parasite examination. Further, the body condition score (BCS) was recorded in the majority of dogs [63]. As the original sample collection was performed by Iwan A. Burgener at the Small Animal Teaching Hospital of the Vetsuisse Faculty, University of Bern, Switzerland, the design of the study was reviewed and approved by the Cantonal Committee of Animal Experimentation, Bern, Switzerland (permit number BE 118/05), and all owners gave written consent prior to inclusion of the dog in the study.

All dogs were assigned a clinical disease severity score (canine IBD activity index [CIBDAI] [64]) both prior to and after initiation of treatment. Each dog in the study was further classified as having signs of either predominantly small intestinal or large intestinal disease, or a combination of both. A gastroduodenoscopy and colonoscopy were performed in each dog enrolled in the study except for four dogs with severe hypoalbuminemia due to protein-losing enteropathy (PLE). In these dogs the preparatory 36-hour fast necessary for colonoscopy was considered potentially harmful, thus the endoscopic examination was limited to a gastroduodenoscopy.

After completion of this standard diagnostic work-up, including gastrointestinal endoscopy with collection of tissue biopsies, all dogs were fed a standardized elimination diet for 14 days. The study diet was a dry single protein diet based on codfish and rice only, with codfish being a novel source of protein for all dogs enrolled in the study. This elimination diet was specially produced for the study (Biomill SA, Granges-Marnand, Switzerland) and was enriched with PUFAs, yielding a concentration of omega-3 PUFAs of 1% and a concentration of omega-6 PUFAs of 3.5% (S1–S3 Files). The adequacy of the diet’s nutritional composition was calculated by a veterinary nutritionist. Owners received detailed instructions on the concept of an elimination diet, strictly prohibiting table scraps and treats other than the prescribed diet. If clinical signs improved significantly or resolved within the first 14 days of feeding the study diet, dogs were assigned to the food-responsive (FRD) group. Dogs that did not improve clinically on the elimination diet were additionally treated with prednisolone (1 mg/kg p.o. BID) for 14 days followed by a slow tapering of the dose. These dogs were allocated to the idiopathic IBD / steroid-responsive disease group. Dogs that did not respond to prednisolone further received cyclosporine (5 mg/kg p.o. SID) or other immunosuppressants (e.g. budesonide 3 mg/m^2^ p.o. SID).

The clinical evaluation after initiation of treatment consisted of a re-evaluation of the CIBDAI score in all dogs and a follow-up gastrointestinal endoscopy in the majority of dogs. The FRD group of dogs was reassessed four weeks after starting the elimination diet, whereas the IBD group of dogs was re-evaluated at 10 weeks after starting treatment with prednisolone.

### Gastrointestinal endoscopy and histopathologic evaluation

Details on the endoscopic and the histopathologic evaluation have been published elsewhere [11]. Briefly, mucosal biopsy specimens were retrieved from the duodenum (approximately 10 cm proximal to the caudal duodenal flexure) and the colon (the middle portion of the descending colon) or from areas with any obvious lesions. Samples were placed in 4% neutral-buffered formalin for 48 hours before being embedded in paraffin and subsequently prepared for histopathologic evaluation.

The endoscopic biopsies were examined histologically by a board-certified pathologist blinded to clinicopathological findings, the number of endoscopy, diagnosis, and treatment. The pathologist assigned a histologic lesion score reflecting the degree of inflammation and cellular infiltration [65].

### Blood sample collection and analysis of the phospholipid profile

During the initial diagnostic work-up and the reassessment of the dogs after initiation of treatment, whole blood and EDTA-plasma samples were collected and immediately stored frozen at -20°C until lipid extraction.

For analysis of the phospholipid profile, 20–200 μL of sample (whole blood or plasma) was mixed with 3.75 volumes of mass spectrometry (MS) grade chloroform:methanol (1:2 [v/v]). After 30 minutes, samples were centrifuged for 5 minutes at 2000×g to remove protein precipitate. The supernatant was then transferred to glass autosampler vials for immediate analysis. Hydrophilic interaction liquid chromatography (HILIC) of polar lipid classes was performed on the extracts as described previously [66,67]. The column effluent was introduced into an Orbitrap Fusion mass spectrometer (MS; Thermo Scientific, Waltham, MA) operated at an orbitrap resolution of 120k for MS1 and with data-dependent MS2 in a linear ion trap. Ion spray ionization was used in the negative mode throughout the entire assay run. Data was converted to mzML format and was analyzed by XCMS v3.00 under R v3.4.2 (R Core Team, 2017. R: A language and environment for statistical computing. R Foundation for Statistical Computing. Available at: https://www.R-project.org/) [68]. Chromatographically obtained m/z peaks were classified based on their retention time and were matched against an *in silico* generated lipid database. Lipid signals were corrected for ^13^C isotope contributions. An exemplary base peak chromatogram with identified phospholipids of one sample is shown as supplementary figure (S1 Fig).

### Statistical analysis

In order to allow for a large number of measured phospholipids and to maintain practicality of the statistical analysis, yet allowing for the detection of significant differences, a data reduction was performed first by means of a principal component analysis (PCA), which was performed with the R package ‘PCAMethods’ (version 1.70.0) using the nonlinear iterative partial least squares (nipals) algorithm with pareto scaling [69]. Principal component analysis was performed on the complete data set, including all identified phospholipid species of both whole blood and plasma samples. These principal components (PrComp) were used as new variables for subsequent analyses. In the full dataset, the majority of phospholipidic variance could be explained by the type of specimen used. Thus, further statistical analysis of the obtained PrComps was performed separately for whole blood and plasma. In order to evaluate the effect of several different variables (including the aforementioned treatment of dogs with FRD or IBD, disease category, interaction of treatment and disease category, age, breed, weight, BCS, and sex) on the variance of the phospholipid profile, the R package ‘LME4’ (version 1.1–17) was used to compare the full linear model with mixed effects to the same model excluding specific factors or interactions. An analysis of variance (ANOVA) was then performed to assess the significance of the effect of a given factor. *P* values < 0.05 were considered statistically significant. The model used for comparisons was chosen according to the lowest Akaike information criterion (AIC) score, and both fixed (treatment, disease category, age, BCS, and weight, including the interaction between treatment and disease category) and random factors (breed, sex, and disease category nested within dog subject) were included in the model. Furthermore, all individual phospholipids were analyzed using the same linear mixed model as for the principal component analysis. The p-values obtained by this method were adjusted for false discovery rate using the Benjamini-Hochberg procedure. In addition, random forest analysis was performed using the web-based program MetaboAnalyst 4.0 to identify phospholipids that possibly contribute to the differentiation between disease category and also between treatment statuses.

## Results

### Animals

Thirty-two dogs were enrolled in the study. Sixteen of the dogs were categorized as FRD, as their clinical signs improved to the extent of being clinically insignificant (CIBDAI score 0–3) after dietary modification. The remaining 16 dogs required additional immunosuppressant treatment based on which these dogs were classified as having IBD. Tables 1 and S1 summarize the characteristics of the dogs enrolled in the study. Nine dogs with IBD (56%) showed hypoalbuminemia and were diagnosed with protein-losing enteropathy as a result of severe lymphoplasmacytic inflammation due to idiopathic IBD. Three of these dogs significantly improved with prednisolone (1 mg/kg p.o. BID) monotherapy, while one dog required treatment with prednisolone and cyclosporine, and another dog required prednisolone and cyclosporine with prednisolone being tapered off and replaced by budesonide later on. Four of the dogs with PLE (44%) were eventually euthanized due to clinical deterioration, with three dogs having received a combination of prednisolone and cyclosporine and one dog having received budesonide and cyclosporine. All other dogs with IBD and normoalbuminemia responded to prednisolone (starting dose 1 mg/kg p.o. BID) which could either be gradually reduced to only a small dose (e.g. 0.25 mg/kg p.o. EOD) or dogs could be taken off of prednisolone completely during the course of treatment. One dog, however, developed severe side effects of prednisolone and was switched to budesonide, which was better tolerated.

**Table 1:** Basic characteristics of the dogs included in the study (n = 32).

Disease	Breed	Age	Sex	Weight	BCS	CIBDAI
IBD, PLE	Papillon	3 y	mn	3.0 kg	3/9	14
IBD, PLE	Rottweiler	10 y	fs	31.6 kg	6/9	9
IBD	Golden Retriever	6 y 10 mo	mn	36.5 kg	6/9	7
IBD	Rottweiler	7 y 7 Mo	f	60.0 kg	8/9	6
IBD, PLE	Beauceron	4 y	fs	28.9 kg	n/a	14
IBD, PLE	Bernese Mountain Dog	5 y	fs	35.5 kg	n/a	12
IBD	American Cocker Spaniel	3 y 7 mo	mn	10.8 kg	5/9	6
IBD, PLE	Yorkshire Terrier/Shi Tzu mix	11 y	fs	4.0 kg	4/9	11
IBD	Mixed breed medium size	12 y 10 mo	mn	27.4 kg	5/9	3
IBD	Cavalier King Charles Spaniel	4 y 6 mo	m	8.6 kg	5/9	4
IBD, PLE	Pug	3 y 6 mo	m	11.2 kg	6/9	8
IBD, PLE	Mixed breed medium size	5 y 11 mo	mn	11.5 kg	3/9	n/a
IBD	Malinois	2 y 8 mo	mn	32.6 kg	4/9	15
IBD, PLE	Labrador mix	3 y	mn	23.2 kg	3/9	5
IBD, PLE	Pug	6 y 10 mo	f	7.2 kg	5/9	4
IBD	Mixed breed medium size	2 y 11 mo	fs	20.3 kg	5/9	4
FRD	Mixed breed medium size	3 y	mn	30.0 kg	7/9	9
FRD	Dachshund	3 y	fs	7.0 kg	4/9	5
FRD	Yorkshire Terrier	8 y 6 mo	fs	2.9 kg	6/9	6
FRD	French Bulldog	1 y 4 mo	m	14.6 kg	5/9	8
FRD	Weimaraner	2 y	fs	23.0 kg	4/9	4
FRD	Tervuren-Irish Wolfshound mix	9 mo	f	25.5 kg	4/9	5
FRD	Samoyed-Border Collie-Swiss Mountain Dog mix	5 y 10 mo	mn	23.0 kg	4/9	7
FRD	Cairn Terrier	3 y	m	9.4 kg	n/a	4
FRD	Golden Retriever	1 y 2 mo	f	20.1 kg	3/9	11
FRD	West Highland White Terrier	1 y	f	6.4 kg	6/9	4
FRD	Labrador Retriever	2 y	m	46.0 kg	6/9	9
FRD	Berger Blanc Suisse	2 y	fs	32.0 kg	n/a	8
FRD	Labrador	11 y 2 mo	mn	32.5 kg	6/9	4
FRD	Mixed breed large size	6 y 2 mo	fs	49.0 kg	7/9	1
FRD	Newfoundland	6 y 9 mo	m	44.2 kg	4/9	4
FRD	German Shepherd Dog	1 y 1 mo	m	34.3 kg	5/9	n/a

The canine inflammatory bowel disease activity index (CIBDAI) refers to the clinical activity score at the first visit.

The body condition score (BCS) refers to the body condition score of the first visit.

y, year; mo, months; f, female; m, male; n, neutered; s, spayed; n/a, not available.

A total of 24 dogs were included in the within-group evaluation of the effect of treatment on the phospholipid profile. In addition to the four dogs with PLE that were euthanized, one dog with FRD was censored from further analysis due to the owners declining a second endoscopy under general anesthesia. Another dog with FRD had to be excluded because of its refusal to eat the study diet, and two additional dogs with IBD were excluded from the study due to one owner declining further participation in the study and the other owner not following the study protocol.

### Phospholipid profiles

**Fig 1:**
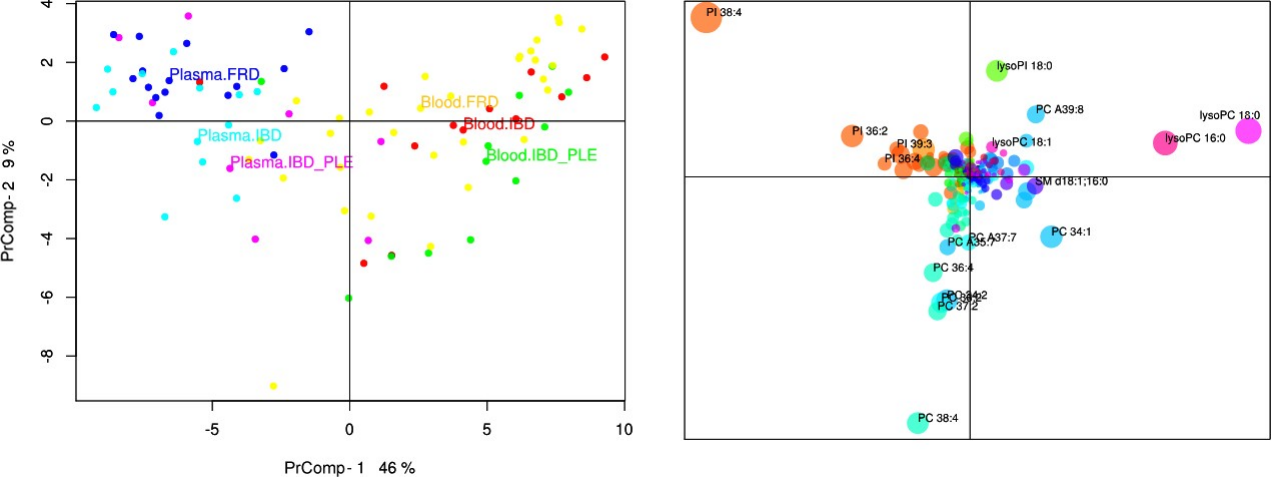
Principal component analysis (PCA) of the phospholipid profile in plasma and whole blood samples. In the score plot (left panel) each dot represents one analyzed sample and similar phospholipid profiles cluster together. A clear separation between plasma and blood samples is observed. In both types of specimen, the three disease categories are similarly positioned to each other, indicating a similar shift in the phospholipid profile in both sample types. Samples are color-coded by sample type and disease classification. The principal component loadings plot (right panel) visualizes the contribution of each phospholipid species to the total variance in the phospholipid profile, to which phospholipid species with the largest distance from the origin contributed the most. Each dot represents a different phospholipid species and the same color is used for the same phospholipid class. Dot sizes are proportional to the MS signal intensity of the phospholipid species. PrComp, principal component; PC, phosphatidylcholine; SM, sphingomyelin; PI, phosphatidylinositol.

Principal component analysis revealed a clear differentiation between the phospholipid profiles of whole blood and plasma regardless of the disease category (Fig 1), thus subsequent analyses were performed separately for whole blood and plasma. Furthermore, as PrComp1, PrComp2 and PrComp3 represented the principal components comprising the majority of the original phospholipid variance, these three PrComps were used for further statistical analysis.

**Fig 2:**
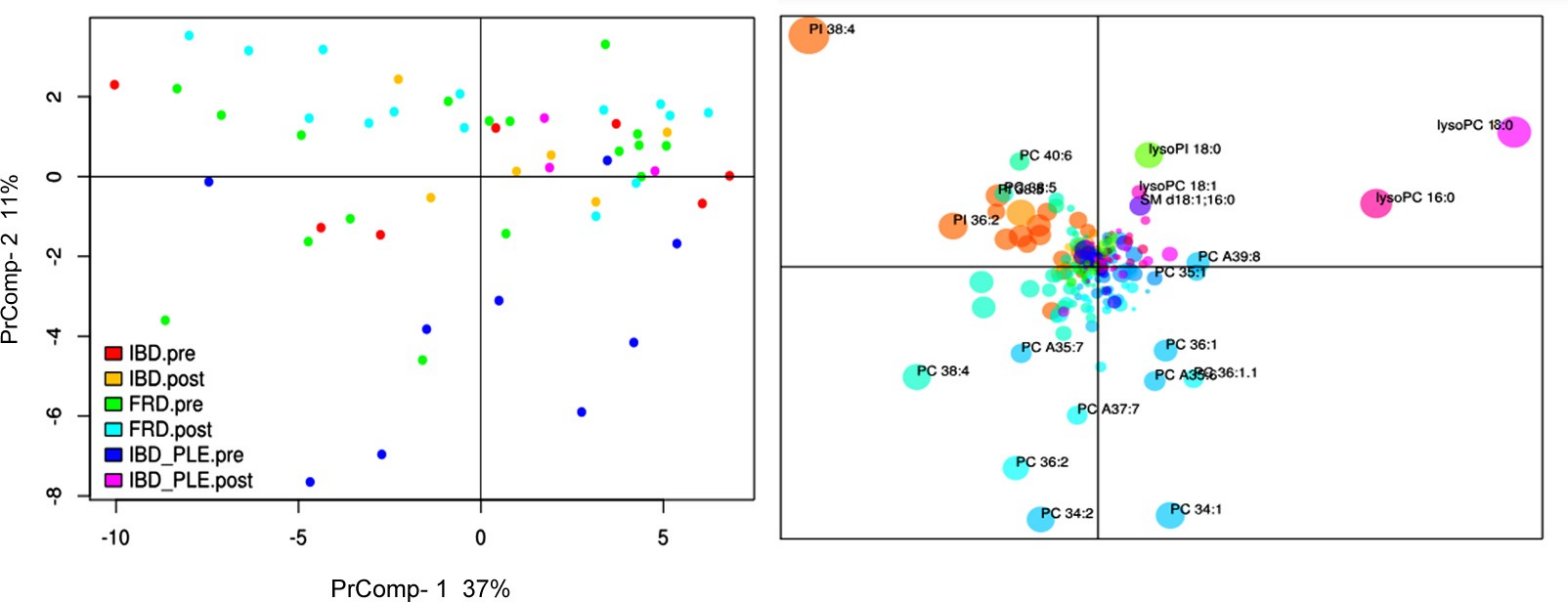
Principal component analysis (PCA) of the phospholipid profile in whole blood samples. In the score plot (left panel, for annotations see Fig 1) samples are color-coded by disease category and treatment status. The post-treatment samples are clustered in the upper right quadrant. In the corresponding loadings plot (right panel) lysolipid species are the predominant phospholipids in the upper right quadrant, indicating that the level of lysolipids increases after treatment. PrComp, principal component; PC, phosphatidylcholine; SM, sphingomyelin; PI, phosphatidylinositol.

**Fig 3:**
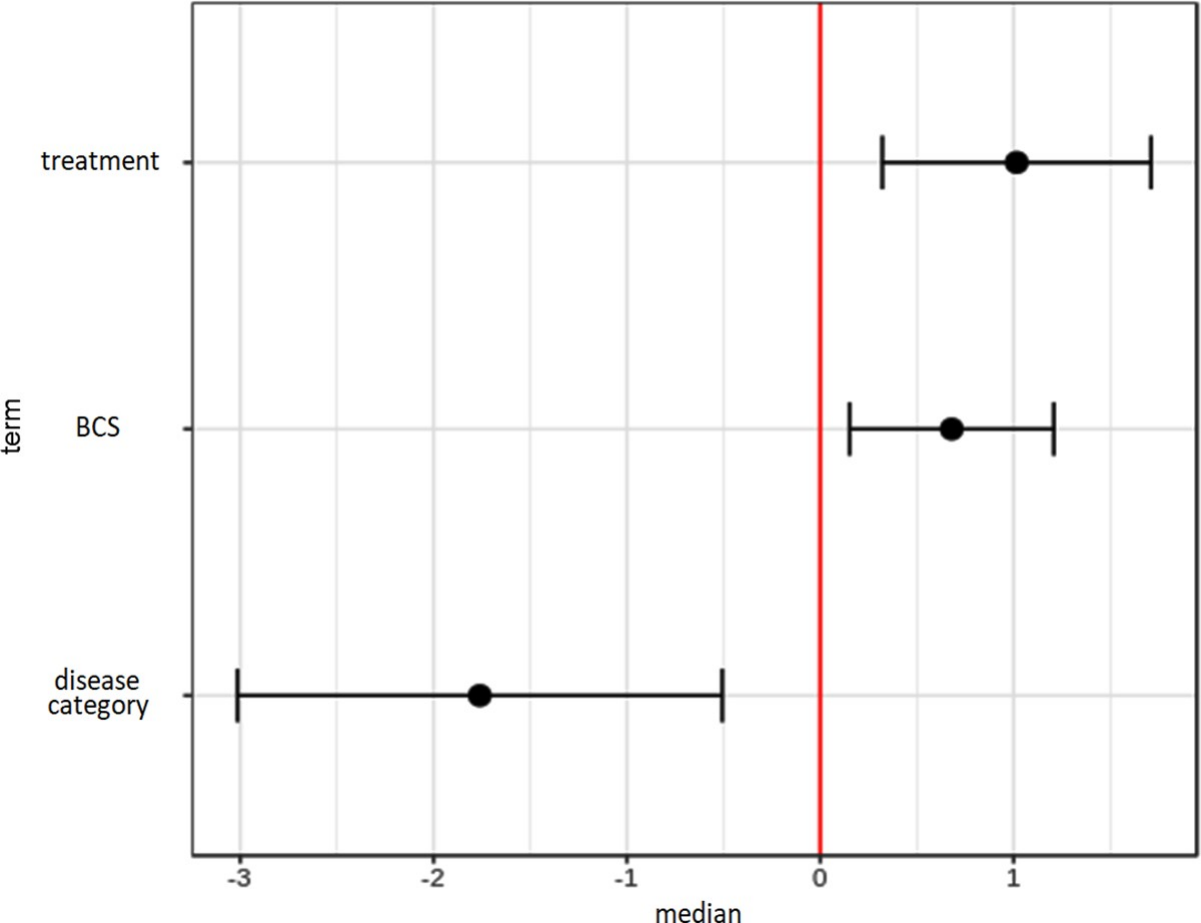
Effect of selected variables on variance of the phospholipid profile on PC2 in whole blood. Fixed effects are plotted with 95% confidence intervals. Treatment, body condition score (BCS), and disease category do not overlap with 0, thus representing significant factors on the variance of the phospholipid composition (ANOVA p < 0.05).

Phospholipid analysis of whole blood did not identify any factors that significantly affected the variance of the phospholipid composition on PrComp1 (Table 2). Despite not reaching significance, breed seemed to have the biggest effect on the variance. However, on PrComp2 the phospholipid profile changed significantly depending on treatment status, with an increase in lysolipids (especially lysophosphatidylcholine [lysoPC] and lysophosphatidylinositols [lysoPI]) after initiation of treatment (Fig 2). Interestingly, after initiation of treatment the originally most abundant phosphatidylcholine (PC) species PC 38:4 (of which the most common molecular species is PC 18:0/20:4) was mainly converted into lysoPC 18:0 and arachidonic acid (i.e. 20:4 fatty acid) (S2 Fig), with a significant change in the ratio of PC 38:4 to lysoPC 18:0 (p < 0.0024, S4 Table). Furthermore, the effect of treatment depended on the disease category, and this effect was largest for dogs diagnosed with PLE due to IBD. Disease category and BCS also showed a significant effect on the phospholipid composition of whole blood in PrComp2 (Fig 3). In contrast, in PrComp3 none of the factors evaluated had a significant effect on the phospholipid profile (Table 2).

**Table 2:** Summary of ANOVA *p*-values of all evaluated parameters.

	*P*-value		
	PrComp1	PrComp2	PrComp3
**Whole blood**			
Interaction treatment & disease category	0.3942	0.5353	0.0828
Treatment	0.7334	**0.0027**	0.2082
Disease category	0.8411	**0.0082**	0.1021
Weight	0.9088	0.1303	0.5920
Age	0.8647	0.3694	0.3668
BCS	0.6296	**0.0296**	0.4735
Breed	0.3097	**0.0081**	1.0000
Sex	1.0000	1.0000	1.0000
**Plasma**			
Interaction treatment & disease category	**0.0332**	**0.0472**	**0.0107**
Treatment	**0.0249**	**0.0238**	**0.0039**
Disease category	0.9593	0.1015	**0.0233**
Weight	0.6662	**0.0075**	**0.0155**
Age	0.1469	**0.0105**	0.5661
BCS	0.1467	0.4361	0.1654
Breed	0.9840	0.1100	1.0000
Sex	1.0000	1.0000	0.6163

PrComp, principal component; BCS, body condition score.

P values < 0.05 considered as significant, highlighted in bold print.

**Fig 4:**
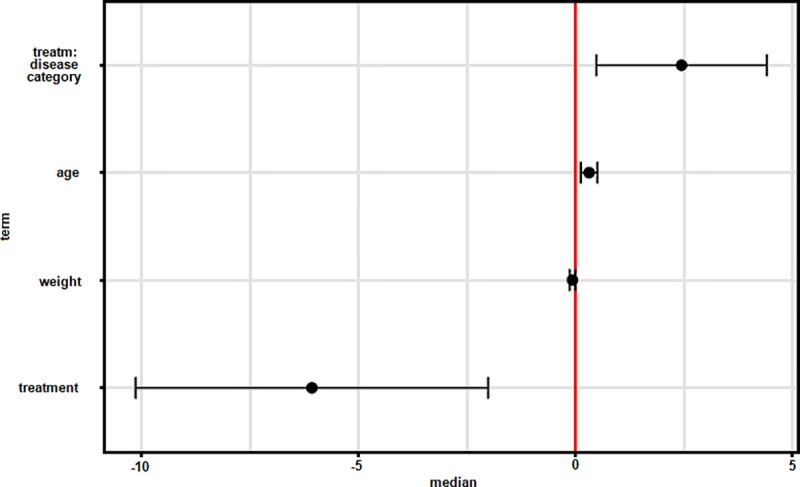
Effect of selected variables on variance of the phospholipid profile on PC2 in plasma. Fixed effects are plotted with 95% confidence intervals. The interaction between treatment and disease category, age, body weight, and treatment do not overlap with 0 and thus significantly affect the variance of the phospholipid composition (ANOVA p < 0.05). treatm:disease category, interaction of treatment and disease category.

The analysis of phospholipids in plasma also revealed significant associations of specific variables and the composition of the phospholipid profile (Table 2). On PrComp1, both treatment and the interaction between treatment and disease category had a significant effect. Similar to whole blood, the most significant effects were detected on PrComp2 in plasma. In addition to treatment and the interaction of treatment with disease classification, age and weight also had an effect on the phospholipid composition (Fig 4). Treatment, disease category, the interaction between treatment and disease category, and body weight were all found to be significant predictors on PrComp3. Sex did not have a significant impact on the phospholipid profile in plasma nor in whole blood samples.

**Fig 5:**
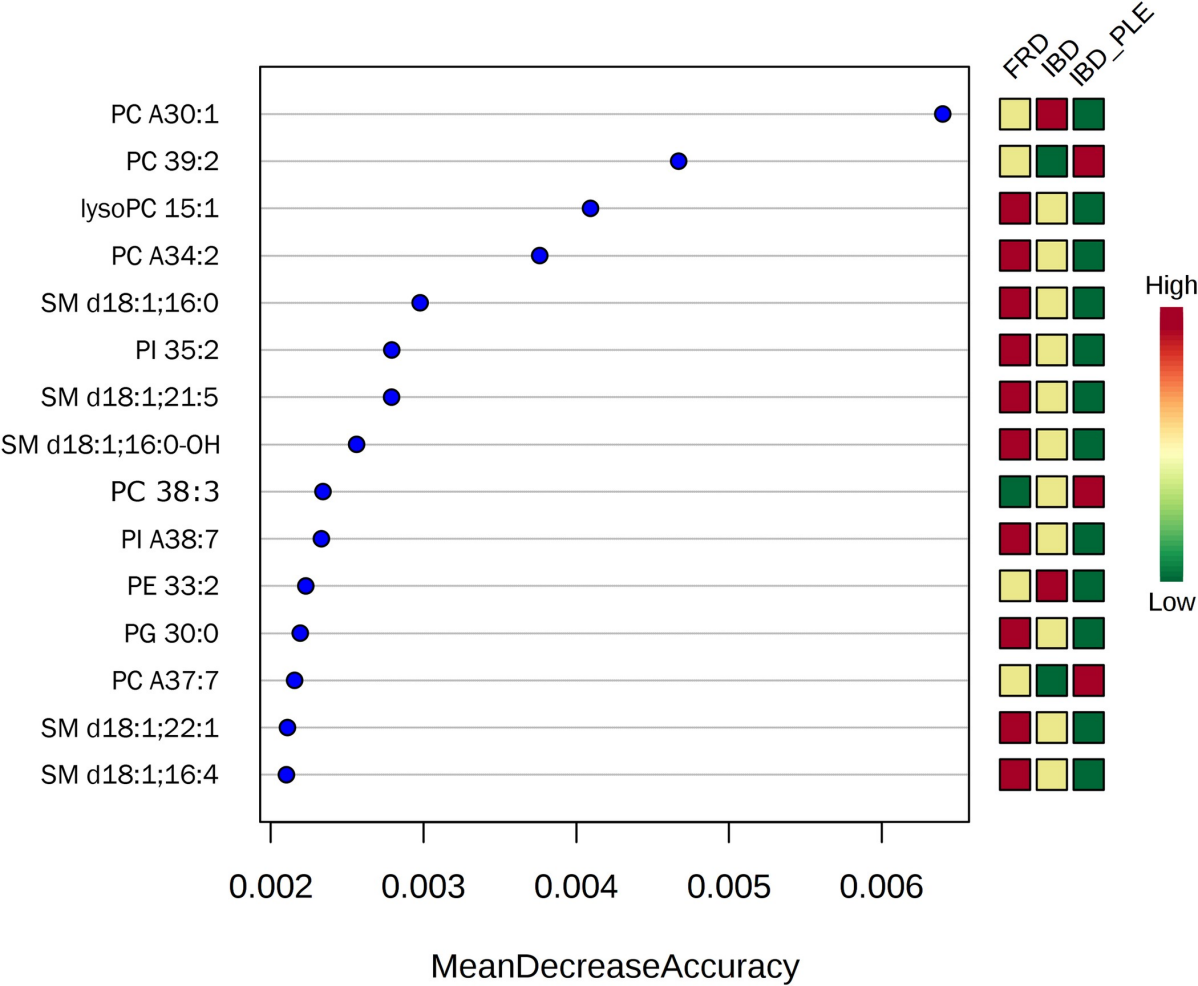
Random forest analysis of phospholipids and their association with disease category. Fifteen phospholipids with the highest discriminatory power between the disease categories are presented. The abundance of the phospholipids is color-coded, with red boxes representing a high abundance and green boxes representing a low abundance of a given phospholipid. PC, phosphatidylcholine; SM, sphingomyelin; PI, phosphatidylinositol; PE, phosphatidylethanolamine; PG, phosphatidylglycerol. 'A' indicates an ether species; the XX:y notation specifies the total number of carbon atoms in the radyl chains ('XX') followed by the number of unsaturation ('y'); for sphingolipids, a sphingosine (d18:1) backbone was assumed.

**Fig 6:**
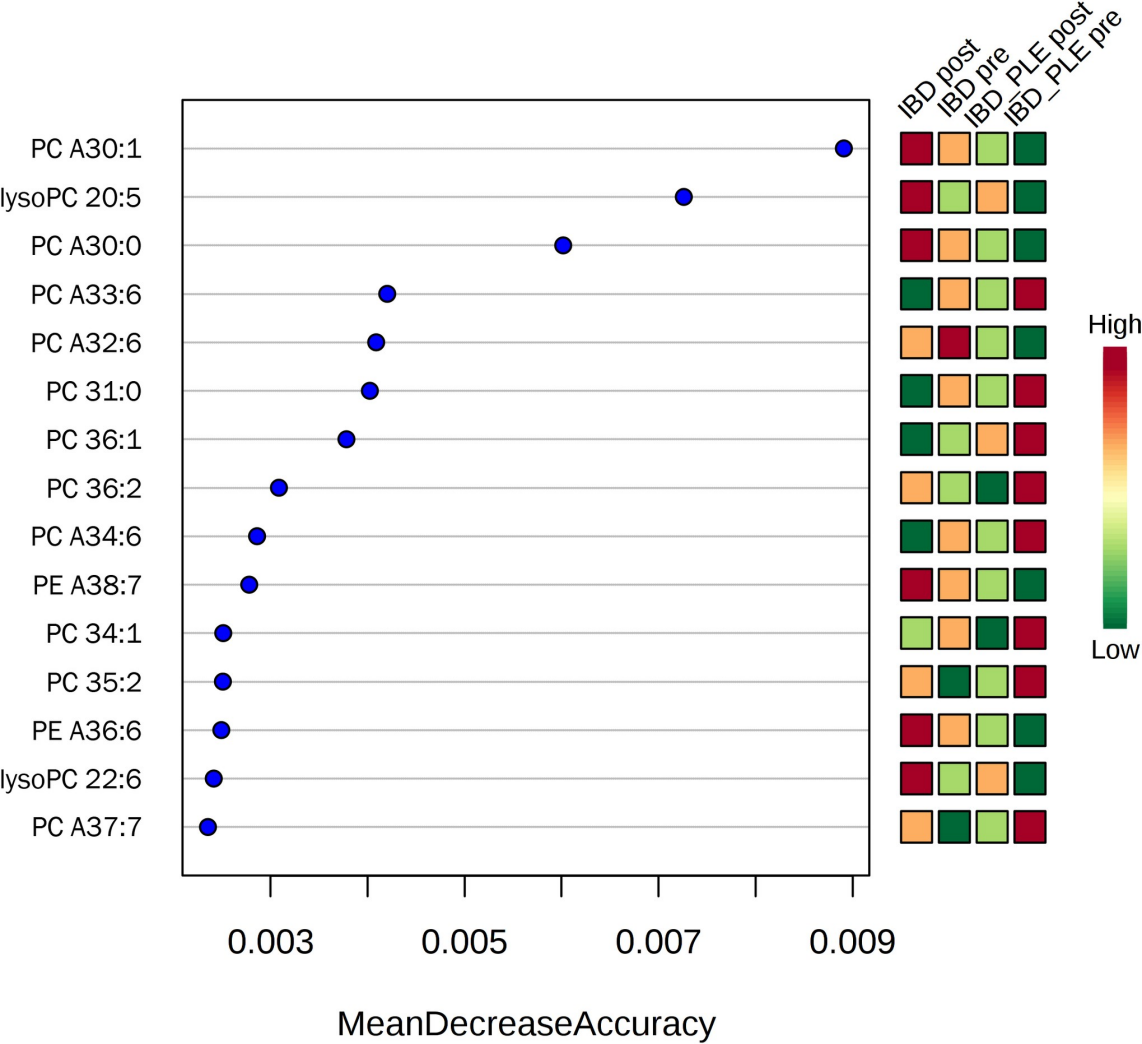
Random forest analysis of phospholipids and their association with treatment in IBD. The 15 top phospholipids based on their importance to discriminate between the treatment statuses of dogs with IBD are shown. Red boxes represent a high abundance, green boxes a low abundance of a given phospholipid. PC, phosphatidylcholine; PE, phosphatidylethanolamine; 'A' indicates an ether species; the XX:y notation specifies the total number of carbon atoms in the radyl chains ('XX') followed by the number of unsaturation ('y').

**Fig 7:**
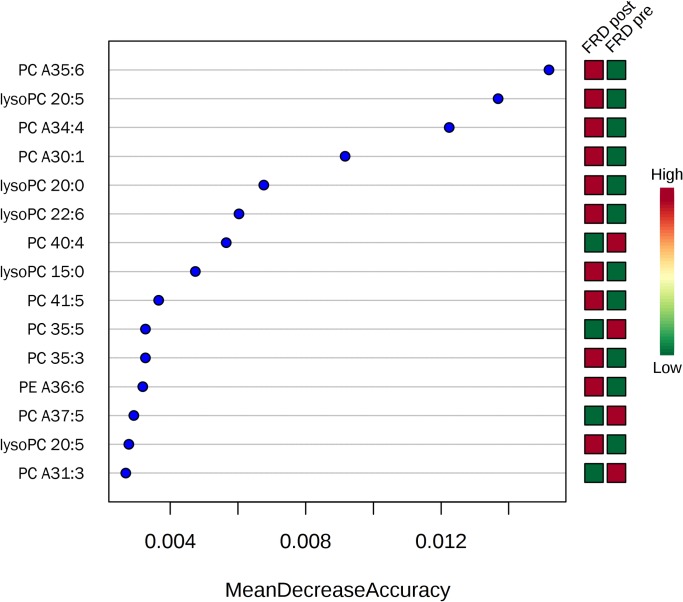
Random forest analysis of phospholipids and their association with treatment in FRD. The 15 top phospholipids based on their importance to discriminate between the treatment statuses of dogs with FRD are shown. Red boxes represent a high abundance, green boxes a low abundance of a given phospholipid. PC, phosphatidylcholine; PE, phosphatidylethanolamine; 'A' indicates an ether species; the XX:y notation specifies the total number of carbon atoms in the radyl chains ('XX') followed by the number of unsaturation ('y').

Random forest analysis revealed several phospholipids with the highest discriminatory power between disease categories and between treatment statuses within one disease category. Figs 5–7 depict the 15 most important phospholipids each that separate either between disease categories (Fig 5) or treatment statuses (Figs 6 and 7).

## Discussion

This is the first study to investigate the systemic phospholipid profile in samples from dogs diagnosed with IBD or FRD and to evaluate changes in the phospholipid profile following the initiation of treatment in these dogs. Significant differences were observed in the phospholipid profiles in dogs with CIE, especially between the two different types of specimen used (whole blood vs. plasma), disease category (IBD vs. FRD), and treatment status. An explanation for the distinct phospholipid profiles in whole blood and plasma samples as discovered by PCA could be the difference in the number of cells within these sample types. As lipids, including phospholipids, are essential components of cellular membranes [70,71], it appears reasonable to expect a difference in the phospholipid composition between whole blood (which contains erythrocytes, leukocytes, and platelets) and plasma (which is essentially devoid of these cells but contains lipoproteins). An influence of different packed cell volumes or numbers of leukocytes of the patients on the findings of this study can currently not be fully excluded. In the current study, slightly more significant effects were found in plasma samples than in whole blood samples. This finding, in addition to a generally better storage stability of plasma [72] as well as less disturbance of the interpretation due to a lower number of unrelated lipids present in the cell membranes, leaves–in the authors’ opinion–plasma the preferable sample type for future studies on the phospholipid profile.

Overall, treatment and disease category were the most significant variables affecting the phospholipid profiles. A significant shift of PC species was detected from PC 38:4 (18:0/20:4) before treatment to the corresponding lysolipid PC 18:0 after treatment. The loss of arachidonic acid (20:4) from the sn-2 position after initiation of treatment might be explained by an activation of phospholipase A2 and thus the liberation of arachidonic acid as a precursor for the synthesis of pro-inflammatory (e.g. prostaglandins and leukotrienes) or anti-inflammatory mediators (e.g. lipoxins and resolvins) [73–75]. Clinical improvement of most of the dogs after treatment would also render a shift towards anti-inflammatory mediators a likely explanation. Furthermore, therapeutic intervention in this study involved an elimination diet based on fish with additional enrichment of PUFAs. PUFAs have been shown to exert several anti-inflammatory actions [47,48,51,76]. Amongst others they decrease the production of arachidonic acid-derived pro-inflammatory eicosanoids (e.g. prostaglandin E_2,_ 4-series leukotrienes) in favor of anti-inflammatory eicosanoids (e.g. prostaglandin E_3,_ 5-series leukotrienes) and they generate further anti-inflammatory mediators such as resolvins and protectins [47,48,75,77]. Thus, the shift to lysoPC 18:0 after treatment might represent an increase in anti-inflammatory mediators, which may be associated with the amelioration of clinical signs observed in the dogs after treatment.

The significant effect of disease category on the phospholipid profile found in this study is interesting and might be due to the severity of the disease. This theory is supported by finding that the significant effect of treatment on the phospholipid profile in whole blood (see above) was linked to disease category, being largest for dogs diagnosed with IBD with PLE. Clinical severity usually increases from FRD to IBD to IBD with PLE [3,5]. Thus, the loss of 20:4 from PC appears to be largest in dogs with the most severe clinical signs. It also appears to be reasonable to assume that the amount of anti-inflammatory mediators required to counteract inflammation is higher with more severe disease. In the current study, disease severity and also clinical improvement were judged using the CIBDAI score. The canine chronic enteropathy clinical activity index (CCECAI), which is an extended scoring index that included the additional criteria hypoalbuminemia, assessment of ascites, peripheral edema and pruritus, was introduced during the current study in 2007 [5], and an attempt was made to use this index for the present investigation. However, as a proper retrospective assessment of the dogs enrolled in this study prior to the CCECAI score’s publication was not possible, and in order to obtain comparable results for all dogs, the CCECAI score was not further assessed in this study.

Age has been shown to affect the lipid profile [78,79]. In this study, dogs with IBD are significantly older than dogs with FRD. Hence, this difference in age could also have contributed to the significant effect of disease category on the phospholipid profile. However, dogs diagnosed with IBD have generally been reported to be older at the time of diagnosis than dogs diagnosed with FRD [3,5] and so, these study dogs represent a typical group of patients in a realistic clinical setting. Although age-matched study groups would have been desirable, the authors still consider the comparison of their representative groups of patients justifiable.

Random forest analysis identified some phospholipids which contribute to the separation of disease category and treatment status. When evaluating the different disease categories, sphingomyelin species were shown to be differentially abundant in dogs with FRD. Sphingomyelin has been reported to promote apoptosis in intestinal epithelial cells and to increase inflammation in mice with induced colitis [80]. The relevant abundance of sphingomyelin species in dogs with FRD could thus result from the inflammatory process of dogs with CIE. Yet, this finding raises the question why sphingomyelin was found to be of importance only in the least severe disease category. The authors, unfortunately, do not have a plausible explanation for this finding at the moment. Also, phosphatidylcholine species were important to differentiate between treatment statuses both in dogs with IBD and FRD. Former studies have demonstrated phosphatidylcholine to possess anti-inflammatory properties and to be beneficial in humans with ulcerative colitis [61,81]. Moreover, patients with ulcerative colitis were reported to have significantly lower levels of phosphatidylcholine and lysophosphatidylcholine compared to healthy controls [82,83]. In the current study, lysophosphatidylcholine species were also significantly relevant to differentiate between treatment statuses in dogs with FRD, with lysophosphatidylcholine species being important after treatment. As all dogs with FRD improved clinically after treatment, this finding is in line with previous studies. However, overall, the true relevance of the phospholipids identified on random forest analysis in the pathogenesis of CIE still remains elusive.

In the current study, the BCS only appeared to influence the phospholipid profile to some degree in whole blood, whereas body weight (but not BCS) presented a significant predictor of the phospholipidic variance in plasma. Previous studies have examined associations between obesity and variations in the lipid profile. While one study found no significant difference in total cholesterol concentrations between obese dogs and lean controls [84], other studies did reveal a clear discrepancy in both cholesterol concentration and lipoprotein profiles when comparing overweight to healthy dogs [79,85]. Mori et al. also suggested that changes in plasma lipoprotein concentrations are more significant in older dogs with obesity (> 8 years of age) and that–as already mentioned above–both age and gender independently affect lipoprotein concentrations. Those findings are also in accordance with other studies [78,86]. In addition, lipid metabolism has been reported to be potentially influenced by breed [78]. The low impact of BCS and body weight on the phospholipid profile observed in the current study might be explained by the fact that the majority of dogs were assigned a BCS between 4/9 and 6/9 at their initial visit, representing a similar and close to ideal BCS in most dogs. Furthermore, as only three dogs were assigned a BCS ≥ 7/9, the influence of obesity could not be evaluated in this study cohort. Also, more advanced diagnostics to determine body fat (e.g. measured by dual-energy x-ray absorptiometry) were not performed in this study. Contrary to previous studies, sex had no effect on the lipid profile in the present study. Neither did the effect of breed reach significance, even though this was the most crucial effect on variance on PrComp1 in whole blood samples. The difference between our findings and those of others might be explained by the focus on different types of lipids. Whereas the current study examined phospholipids, former studies have mostly investigated concentrations of cholesterol and lipoproteins.

The lack of a control group presents a limitation of this study. However, previous studies have already evaluated the lipid profile in healthy dogs [84,87–91] and the main objective of the current study was to describe and compare the phospholipid profile of dogs with IBD or FRD before and after treatment. Nevertheless, a control group of dogs with CIE receiving the same treatment but short of PUFAs would have allowed for assessing the sole effect of supplemental PUFAs on the phospholipid profile and the dogs’ clinical response. The stability of fatty acids during storage poses an additional limitation, as recent studies have reported fatty acids to be affected by degradation during long-term storage and have revealed storage at -80°C to be the most stabilizing [72,92–94]. In this study, all samples had been stored frozen at -20°C for approximately 10 years without discontinuity of the cooling chain. To the authors’ knowledge, the exact mechanisms and kinetics of phospholipid degradation over a period of ten years have not been reported, but a similar risk of degradation is most likely for the current samples because all samples were subjected to the exact same storage conditions. An increase in lysolipids due to degradation cannot be excluded in this study, but the effect of storage on the phospholipid profile represents a methodic error, leaving–in the authors’ opinion–the findings of this study (e.g. significant variance of the phospholipid profile depending on disease category) still reliable. Another shortcoming is the small study cohort of our investigation. A larger number of dogs might have revealed a more distinct phospholipid signature in each disease group and with response to treatment. However, the number of dogs in this study was clearly affected by both the ethical aspect of performing a repeat endoscopy in dogs that had clinically improved as well as the willingness of the owners to consent to repeat examinations.

In addition, a confounding effect of the different time period between pre- and post-treatment evaluation in the two disease groups cannot be excluded in this study. However, as the treatment with immunosuppressant medication in dogs with IBD was started 14 days after the dietary modification and based on the absence of clinical improvement on the diet alone, those dogs had to be allowed more time to respond to treatment. Clinical signs in dogs with FRD typically improve faster on appropriate treatment, while dogs needing immunosuppressant treatment can take longer to respond. Thus, the different time scales were necessary to ensure correct classification of the disease status and were also chosen according to previous studies [5,11,62].

The duration of the elimination diet trial was set to 14 days. Potentially, few dogs with FRD could have not yet responded within that time period. However, this duration was chosen for the dietary trial according to current consensus that dogs with FRD typically show significant clinical improvement within 14 days of a strict dietary trial [3,5,95,96]. In addition, this time frame assisted with proper owner compliance. Similarly, few dogs with IBD may have improved on the diet at first and thus, could have been incorrectly classified as FRD. However, despite dietary modification being important in the management of canine IBD, it is the authors’ experience that dogs with IBD respond only to a certain extent to an elimination diet alone. Thus, it appears to be unlikely that dogs with IBD showed either significant improvement or clinical remission on the elimination diet resulting in incorrect classification.

The histopathologic score was assigned in this study according to a publication by Jergens et al. [65]. The World Small Animal Veterinary Association (WSAVA) Gastrointestinal Standardization Group released a new guideline for histopathological evaluation of gastrointestinal tissues in 2008 [97], which was revised and released as an ACVIM consensus statement in 2010 [10]. Similar to the extended clinical scoring system (CCECAI [5]), the 2008 WSAVA gastrointestinal histopathology guideline was not adopted during the course of the current study in order to apply the same histopathologic criteria to all dogs included.

Glucocorticoids, regardless of endogenous or exogenous excess, have been recognized to cause hyperlipidemia in dogs, mainly reflected in a mild hypercholesterolemia and hypertriglyceridemia and increased very low density lipoproteins [38,84,86,98,99]. Similarly, cyclosporine has been reported to cause hyperlipidemia characterized by hypercholesterolemia and increased serum concentrations of low density lipoprotein cholesterol [100–102]. Furthermore, glucocorticoids are known to modulate arachidonic acid metabolism and alter membrane phospholipids [103,104]. Hence, an effect of immunosuppressant medication on the phospholipid profile cannot be excluded. As immunosuppressants are crucial in the treatment of canine IBD, this influence on the results could not be avoided. Comparison of the findings in this study to the plasma phospholipid composition in a healthy cohort receiving the study diet together with an immunosuppressant could have helped distinguishing the effects of the disease from those of the immunosuppressant on the plasma phospholipid profile. However, the use of immunosuppressant medications in healthy pet dogs was considered unethical by the authors and would have been also very difficult to get approved by the local ethics committee.

Finally, several studies have concluded that diet composition can considerably affect lipid metabolism and cholesterol concentrations [78,85]. A study by Jeusette et al. revealed that a nutritional modification to a high-protein low-energy diet had advantageous effects on plasma lipids in obese dogs [85]. Pasquini et al. described, amongst other findings, that serum cholesterol concentrations were the lowest in dogs fed a diet with a high content of fish. Also, diet composition has been shown to impact the phospholipid composition and characteristics of cell membranes (e.g. lipid rafts, membrane fluidity), rendering dietary phospholipids a potential therapeutic avenue [49,105,106]. Furthermore, an effect of the diet given prior to inclusion of a dog in the study on the pre-treatment systemic phospholipid profile remains a possibility. The dogs received a large variety of different diets including both dry and canned diets of various brands as well as home-made diets before entering the study. A standardization of the dogs’ diets prior to enrollment in this study would have been extremely difficult if not impossible due to the variety of previous diets as well as the medical situation of the dogs presented, often showing severe clinical symptoms and the owner’s understandably strong desire to obtain timely medical treatment–without first standardizing the patient’s diet for a longer period of time. Hence, a meaningful comparison of the nutritional content of previous diets and the study diet would have been ideal, but unfortunately was not feasible given the medical conditions of the dogs in this study.

In summary, this is the first study to analyze the systemic phospholipid profile in dogs with IBD or FRD before and also after treatment. The severity of the disease and the effect of treatment most significantly determined the composition of the phospholipid profile, and a significant shift in the phospholipid species was observed after treatment (PC 38:4 to lysoPC 18:0). These findings suggest that the phospholipid profile is an informative tool and might have clinical utility for evaluating the response to treatment. Future studies with improved sample storage conditions are warranted to verify the results of this study in a larger group of dogs, to further investigate the association of different lipids with the pathophysiology of canine CIE, and to evaluate their potential as a novel therapeutic approach to canine IBD and FRD.

## Supporting information

S1 Fig Base peak chromatogram of one of the samples. Base peak chromatogram of the separation by hydrophilic interaction liquid chromatography of phospholipids extracted from blood of a dog with FRD before treatment. Colored dots indicate retention time and m/z ratio of phospholipids detected by orbitrap ultrahigh resolution mass spectrometry.
(TIF)

S2 Fig Effect of treatment on the individual phospholipids. The dashed red horizontal line is located at p = 0.05, with dots above the line having p-values < 0.05; p values < 0.05 considered as significant.
(PDF)

S1 Table Characteristics of the dogs with IBD (n = 16) or FRD (n = 16) included in the study. (DOCX)

S2 Table Raw peaklist of annotated phospholipids. Raw peaklist including the numbers of all 331 phospholipid signals in the samples analyzed. Phospholipids were annotated based on retention time and mass to charge (m/z) ratio. Phospholipids annotated with an '*' had a difference between theoretical and observed m/z of > 0.015 Da (but < 0.050) and should be considered 'tentatively identified'. Retention times and observed m/z values are included in the peaklist. Disease category: red = IBD; blue = IBD with PLE; green = FRD.
(XLS)

S3 Table Sample identification and patient information. (XLS)

S4 Table P-values of effect of treatment, disease category and sample type on individual phospholipids. (XLSX)

S1 File Nutritional composition of the study diet. (DOC)

S2 File Table of nutritional content (original in French). (DOC)

S3 File Results of external PUFA analysis by Swiss reference laboratory (original in French). (PDF)
