# Supplementary figures and images for: Comparison of the systemic phospholipid profile in dogs diagnosed with idiopathic inflammatory bowel disease or food-responsive diarrhea before and after treatment
Source: PLoS One. 2019 Apr 16;14(4):e0215435. doi: 10.1371/journal.pone.0215435 (PMC6467395; doi:10.1371/journal.pone.0215435)

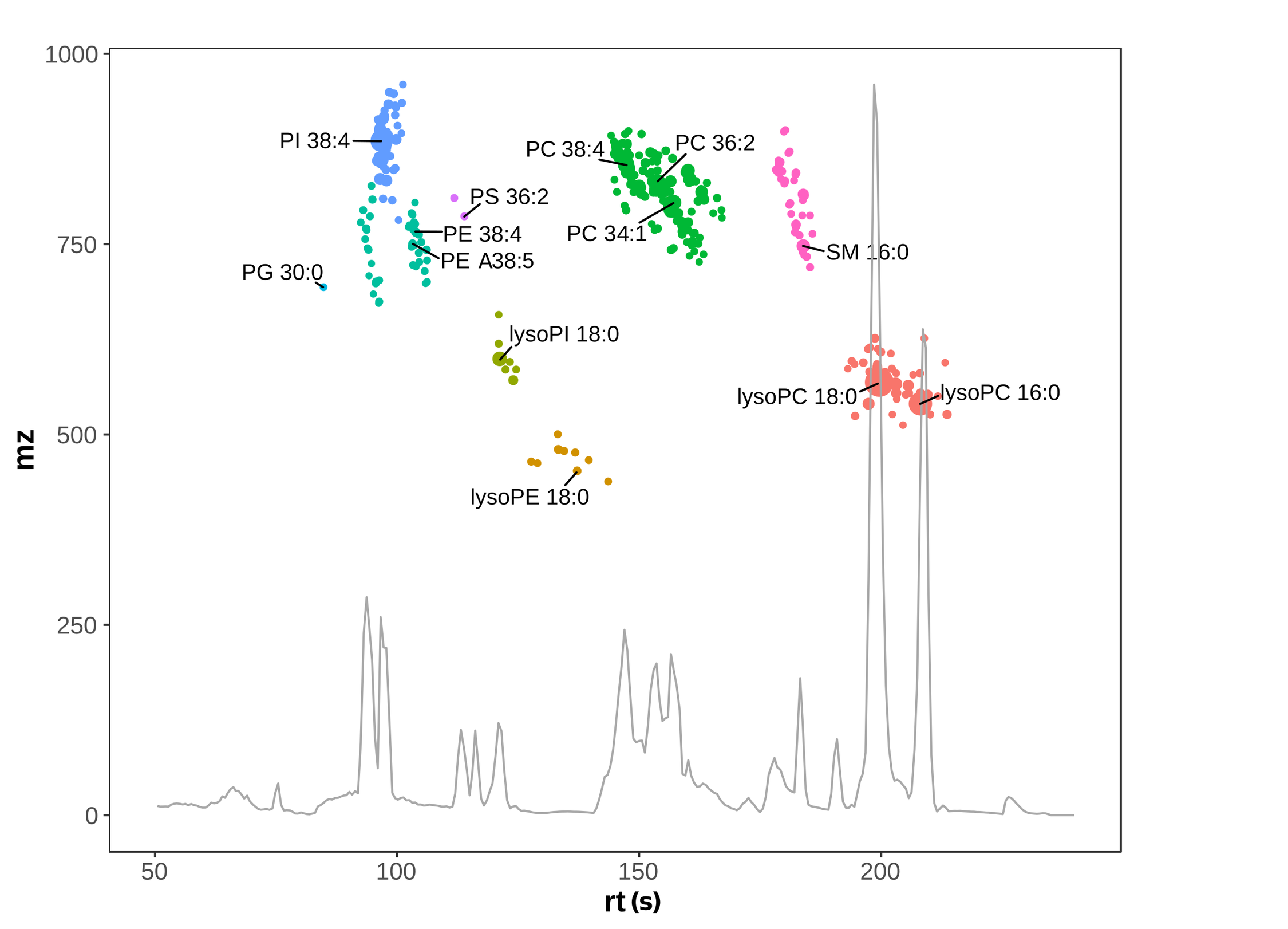

Supplement: S1 Fig — Base peak chromatogram of the separation by hydrophilic interaction liquid chromatography of phospholipids extracted from blood of a dog with FRD before treatment. Colored dots indicate retention time and m/z ratio of phospholipids detected by orbitrap ultrahigh resolution mass spectrometry. (TIF) [file pone.0215435.s001.tif]

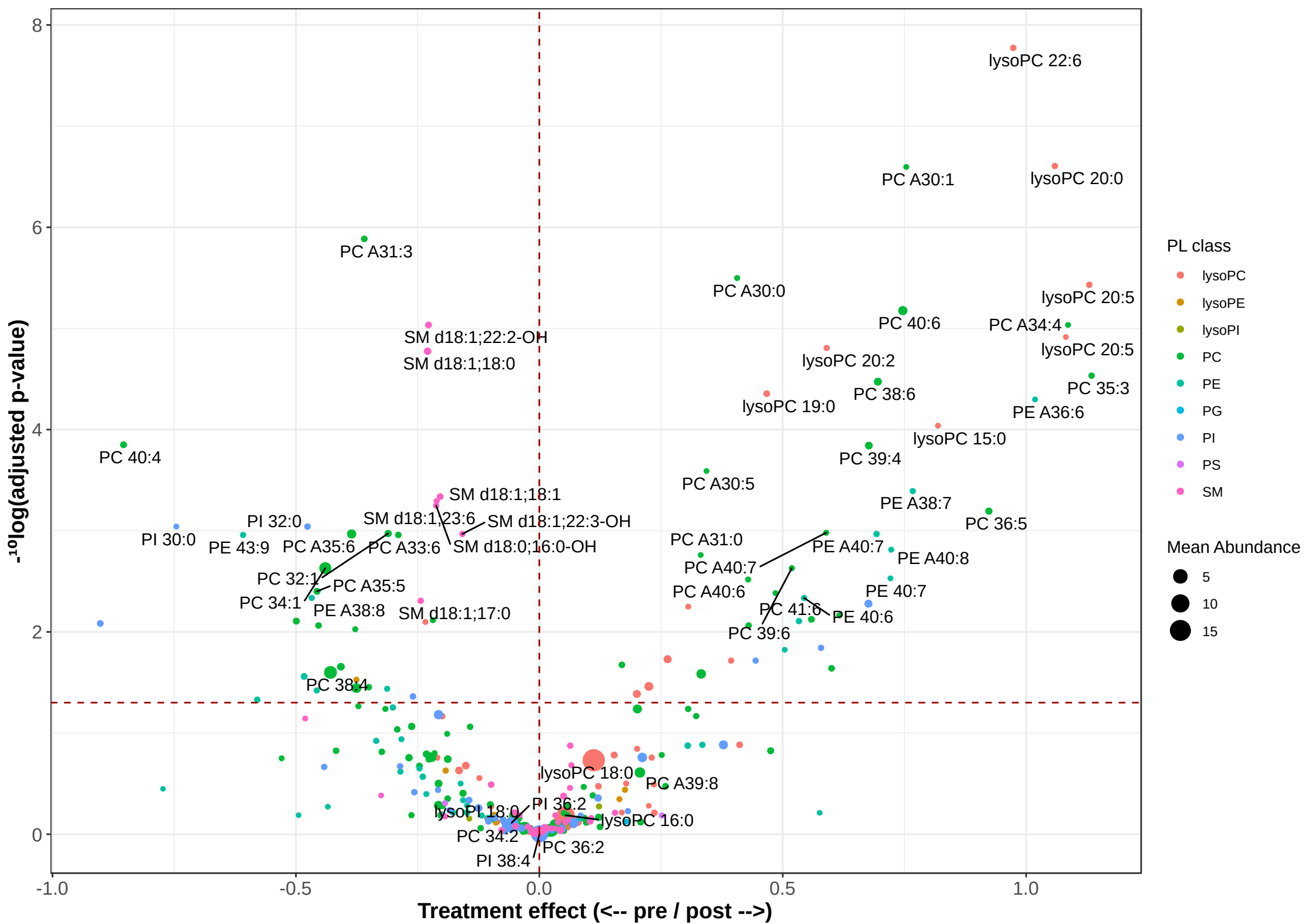

Supplement: S2 Fig — The dashed red horizontal line is located at p = 0.05, with dots above the line having p-values < 0.05; p values < 0.05 considered as significant. (PDF) [file pone.0215435.s002.pdf]
