# Supplementary material for: Comparison of the systemic phospholipid profile in dogs diagnosed with idiopathic inflammatory bowel disease or food-responsive diarrhea before and after treatment
Source: PLoS One. 2019 Apr 16;14(4):e0215435. doi: 10.1371/journal.pone.0215435 (PMC6467395; doi:10.1371/journal.pone.0215435)
Supplement: S1 Table — (DOCX) [file pone.0215435.s003.docx]

**S1 Table. Characteristics of the dogs with IBD (n=16) or FRD (n=16) included in the study**.

| **Group characteristic** | **IBD** | **FRD** | ***P*-value** |
| --- | --- | --- | --- |
| Total number | 16 | 16 | **-** |
|  | |  |  |
| Age in years, median (IQR) | 4.8 (3.1–7.4) | 2.5 (1.2–6.1) | **0.020*** |
| Sex, male/female | 9/7 | 8/8 | 0.723** |
| Body weight in kg, median (IQR) | 21.8 (9.2–32.4) | 24.3 (10.7–33.9) | 0.665* |
| Body condition score, median (IQR) | 5 (4–6) | 5 (4–6) | 0.638* |
| Breed, n (%) |  |  | 0.694** |
| - Pure-bred dogs | 11 (69%) | 12 (75%) |  |
| - Mixed breed dogs | 5 (31%) | 4 (25%) |  |
| CIBDAI score, median (IQR) | 7 (4–12) | 5 (4–8) | 0.201* |

∗*P-*values obtained by Wilcoxon rank-sum test. *P-*values *<* 0.05 considered as significant.

***P*-values obtained by likelihood ratio test for association. *P-*values *<* 0.05 considered as significant.
