# Supplementary material for: Comparison of the systemic phospholipid profile in dogs diagnosed with idiopathic inflammatory bowel disease or food-responsive diarrhea before and after treatment
Source: PLoS One. 2019 Apr 16;14(4):e0215435. doi: 10.1371/journal.pone.0215435 (PMC6467395; doi:10.1371/journal.pone.0215435)
Supplement: S1 File — (DOC) [file pone.0215435.s007.doc]

**S1 File. Nutritional composition of the study diet.**

Adult Sensitive Gastrointestinal

Codfish and Rice *

| AVERAGE ANALYSIS |  | MINERALS |  |
| --- | --- | --- | --- |
| Moisture | 8 % | Calcium | 1.1 % |
| Crude protein | 24 % | Phosphorus | 0.8 % |
| Crude fat | 13 % | Potassium | 0.54 % |
| Crude ash | 6.5 % | Sodium | 0.4 % |
| Crude fiber | 2.5 % | Magnesium | 0.1 % |
| Carbohydrates (NfE) | 45 % | Iron | 125 mg/kg |
| *Dietary fibers* | 6 % | Copper | 15 mg/kg |
| Starch |  | Manganese | 20 mg/kg |
|  |  | Zinc | 200 mg/kg |
|  |  | Iodine | 2 mg/kg |
|  |  | Selenium | 200 mcg/kg |
|  |  |  |  |
|  |  |  |  |
| VITAMINS |  | FATTY ACIDS |  |
| Vitamin A | 13000 UI | Fatty acids (Omega 6) | 3.5 % |
| Vitamin D3 | 1500 UI | Fatty acids (Omega 3) | 1.0 % |
| Vitamin E | 100 mg |  |  |
| Vitamin B1 | 10 mg |  |  |
| Vitamin B2 | 20 mg | ENERGY | /kg |
| Pantothenic acid | 30 mg | Metabolizable E. (Atwater) | 3939 kcal |
| Niacin (PP) | 40 mg | Metabolizable E. (AAFCO) | 3520 kcal |
| Vitamin B6 | 8 mg | Metabolizable E. (NCR) | 3680 kcal |
| Vitamin B12 | 100 mcg |  |  |
| Biotin | 0.8 mg |  |  |
| Folic acid | 2 mg | **SPEC.** |  |
| Choline | 1900 mg | L-Carnitine | 100 mg/kg |
| Biocholine | 225 mg | DL-Methionine | 330 mg/kg |
|  |  |  |  |

Biomill Adult Sensitive Gastrointestinal is a quality product made in Switzerland, developed in cooperation with the University of Bern and distributed by Biomill AG, 1523 Granges-Marnand (Switzerland) [www.biomill.ch](http://www.biomill.ch/) FREECALL 0800 554 310 only in Switzerland

|  | 22.08.08 DIs |
| --- | --- |
