# Supplementary material for: Comparison of the systemic phospholipid profile in dogs diagnosed with idiopathic inflammatory bowel disease or food-responsive diarrhea before and after treatment
Source: PLoS One. 2019 Apr 16;14(4):e0215435. doi: 10.1371/journal.pone.0215435 (PMC6467395; doi:10.1371/journal.pone.0215435)
Supplement: S3 File — (PDF) [file pone.0215435.s009.pdf]

STS 072
ISO 17025
GMP

UFAG LABORATORIEN AG
Kornfeldstrasse 4
Postfach
CH-6210 Sursee

Telefon +41 (0)41 926 83 30
Telefax +41 (0)41 926 83 40
e-mail info@ufag-laboratorien.ch
www.ufag-laboratorien.ch

UFAG LABORATORIEN
Untersuchung und Forschung

Client: Moulins de Granges SA, Mme Valérie Vincent, Granges-Marnand
Liste de distribution: Moulins de Granges SA, Mme Valérie Vincent, Granges-Marnand
Facture: Moulins de Granges SA, Granges-Marnand

Rapport

Rapport No.: 07-23945

Reçu le: 24.12.07 Terminé le: 10.01.08

Sommaire

Pos.	Numéro d'éch.	Nom, Désignation
1	07-23945-001	Diet low (Art. 89C)

Sursee, 10.01.2008

Votre interlocuteur (-trice):

Susanne Täuber
Leiterin Analytik Lebensmittel

Digitally signed by www.ufag-laboratorien.ch
Date: 2008.01.10 16:20:49 CET
Reason: Unterschrieben von Stephanie Schuster
Location: CH-6210 Sursee

Ce rapport a été généré par un système informatique des laboratoires validé.
La libération a été effectuée par une signature électronique traçable.

Les résultats obtenus se réfèrent exclusivement aux échantillons examinés. Sur demande, le client peut obtenir des informations plus précises quant aux méthodes d'investigation utilisées. Les méthodes suivies du signe [*] n'entrent pas dans le champ d'applications de l'accréditation. Les méthodes suivies du signe [**] sont sous-traitées dans un autre laboratoire. Ce rapport d'analyses ne peut faire l'objet d'une reproduction partielle sans l'autorisation écrite des Laboratoires UFAG. Nos conditions générales en vigueur sont applicables.

STS 072
ISO 17025
GMP

UFAG LABORATORIEN AG
Kornfeldstrasse 4
Postfach
CH-6210 Sursee

Telefon +41 (0)41 926 83 30
Telefax +41 (0)41 926 83 40
e-mail info@ufag-laboratorien.ch
www.ufag-laboratorien.ch

UFAG LABORATORIEN
Untersuchung und Forschung

Rapport

Rapport No.: 07-23945

Reçu le: 24.12.07 Terminé le: 10.01.08

Client: Moulins de Granges SA, KdNr. 15490
Mme Valérie Vincent
CH-1523 Granges-Marnand

Position: 1
Numéro d'éch.: 07-23945-001
Nom: Diet low (Art. 89C)
Note: Prod.: 20.12.2007

Méthode, technique de mesure

Objet d'analyse		Résultat	Unité	Valeur réf.	Valeur tol.	Valeur limite	LDT / LD
**Nutritives**							
MSDA; gravimétrique (hydr. acide)							
Matières grasses		16.9	g/100g				
**Acides gras**							
MSDA; GC-FID							
A.caproique	C 6	ndt	g/100g				0.1
A.caprylique	C 8	ndt	g/100g				0.1
A.caprique	C 10	ndt	g/100g				0.1
Acide laurique	C 12	ndt	g/100g				0.1
Acide myristique	C 14	0.2	g/100g				
Acide palmitique	C 16	3.2	g/100g				
Acide palmitoléique	C 16:1	0.5	g/100g				
Acide margarique	C 17	ndt	g/100g				0.1
Acide stéarique	C 18	0.9	g/100g				
Acide oléique	C 18:1	5.4	g/100g				
Acide linolique	C 18:2	3.9	g/100g				
alpha-acide linolénique	C 18:3	0.4	g/100g				
gamma-acide linolénique	C 18:3	ndt	g/100g				0.1
A.arachidique	C 20	ndt	g/100g				0.1
Acide gadoléique	C 20:1	0.1	g/100g				
A.eicosadienoï	C 20:2	ndt	g/100g				0.1
A.eicosatrién.	C 20:3	ndt	g/100g				0.1
A.arachidonique	C 20:4	0.1	g/100g				
A.eicosapentaé	C 20:5	0.2	g/100g				
A.béhénique	C 22	ndt	g/100g				0.1
Acide erucique	C 22:1	ndt	g/100g				0.1
A.docosadiénoi.	C 22:2	ndt	g/100g				0.1
A.docosatétraé.	C 22:4	ndt	g/100g				0.1
A.docosapentaé.	C 22:5	0.1	g/100g				
A.docosahexaé.	C 22:6	0.3	g/100g				
Acide lignocérique	C 24	ndt	g/100g				0.1
Acide sélacholéique	C 24:1	ndt	g/100g				0.1
Acide octadécatétr.	C 18:4	ndt	g/100g				0.1
**Acides gras totales**							
Acide gras saturés		4.3	g/100g				
Acides gras mono-insat.		6.0	g/100g				

Légende: nd = non détectable (inférieure à la LD) LD = Limite de détection UFC = Unités formant colonie
ndt = non déterminable (inférieure à la LI LDT = Limite de détermination MS = Matière sèche

STS 072
ISO 17025
GMP

UFAG LABORATORIEN AG
Kornfeldstrasse 4
Postfach
CH-6210 Sursee

Telefon +41 (0)41 926 83 30
Telefax +41 (0)41 926 83 40
e-mail info@ufag-laboratorien.ch
www.ufag-laboratorien.ch

UFAG LABORATORIEN
Untersuchung und Forschung

Rapport

Rapport No.: 07-23945

Reçu le: 24.12.07 Terminé le: 10.01.08

Client:	Moulins de Granges SA, KdNr. 15490<br>Mme Valérie Vincent<br>CH-1523 Granges-Marnand
Position:	1
Numéro d'éch.:	07-23945-001
Nom:	Diet low (Art. 89C)

Méthode, technique de mesure

Objet d'analyse	Résultat	Unité	Valeur réf.	Valeur tol.	Valeur limite	LDT / LD
**Acides gras totales**						
MSDA; GC-FID						
Acides gras polyinsat.	5.0	g/100g				
Ac. gras polyins. oméga-3	1.0	g/100g				
Ac. gras polyins. oméga-6	4.0	g/100g				

Légende:
nd = non détectable (inférieure à la LD) | LD = Limite de détection | UFC = Unités formant colonie
ndt = non déterminable (inférieure à la LI | LDT = Limite de détermination | MS = Matière sèche
