# Supplementary material for: Comparison of the systemic phospholipid profile in dogs diagnosed with idiopathic inflammatory bowel disease or food-responsive diarrhea before and after treatment
Source: PLoS One. 2019 Apr 16;14(4):e0215435. doi: 10.1371/journal.pone.0215435 (PMC6467395; doi:10.1371/journal.pone.0215435)
Supplement: Supplementary file 8 [file pone.0215435.s008.doc]

Edition Recettes en colonne

Groupe	Blum	Dossier	Fab
Liste de Prix	2008 net	Jeu	totales

	**Etab. :**<br>**Formule :**<br>**N° Ordre :**<br>**N° Optim :**		**05**<br>**347**<br>**5031**<br>**8571**	**05**<br>**890**<br>**5154**<br>**8070**	**05**<br>**891**<br>**5155**<br>**8054**
	Prix de la Recette		92.90	114.39	122.80
0001	POIDS	100	100.00	100.00	100.00
0002	MS	%	88.71	90.50	90.50
0003	PROTEINE	%	23.74	26.65	26.65
0004	GRAISSE	%	13.01	16.51	16.51
0004	C18 2 n6	%	2.72	3.86	3.12
0004	C18 3 n3	%	0.58	0.74	1.37
0005	CELL	%	2.13	2.12	2.12
0005	FIBRES	%	6.31	6.74	6.74
0006	MIN cendres	%	6.37	7.77	7.77
0007	NfE	%	43.46	37.45	37.45
0007	GE	KJ/	1816.50	1919.11	1919.11
0007	sV GE	%	88.46	88.48	88.48
0008	Energy NCR	KJ/	1516.51	1600.09	1600.09
0009	Energie AAFCO	KJ/	1447.21	1526.36	1526.36
0009	Energie AAFCO	CA	345.75	364.65	364.65
0011	LYS	%	1.48	1.87	1.87
0012	MET	%	0.48	0.65	0.65
0013	CYS	%	0.44	0.43	0.43
0014	M+C	%	0.93	1.10	1.10
0015	THR	%	1.00	1.17	1.17
0016	TRP	%	0.25	0.28	0.28
0017	ARGIN	%	1.22	1.41	1.41
0018	HISTID	%	0.53	0.61	0.61
0019	ISOLEU	%	1.11	1.29	1.29
0020	CARNITI	Gr	0.10	0.10	0.10
0029	CA	%	1.09	1.28	1.28
0030	PHOS	%	0.82	0.92	0.92
0031	PHOD	%	0.74	0.75	0.75
0032	K	%	0.52	0.58	0.58
0033	NA	%	0.41	0.43	0.43
0034	CL	%	0.66	0.70	0.70
0035	MAGNE	%	0.10	0.11	0.11
0036	FER	Gr	0.10	0.10	0.10
0037	CU	Gr	0.01	0.01	0.01
0038	MANGAN	Gr	0.02	0.02	0.02
0039	ZINC	Gr	0.20	0.20	0.20
0040	IODE	Gr	0.00	0.00	0.00
0041	SELENIUM	mg	0.20	0.20	0.20
0042	COBALT	mg	0.12	0.11	0.11
0039	ZINC org.	Gr	0.05	0.05	0.05
0045	VIT. A	Kui	13.40	13.40	13.40
0046	VIT. D3	Kui	1.50	1.50	1.50
0047	VIT. E	Gr	0.10	0.10	0.10
0050	VIT. B1	Gr	0.01	0.01	0.01
0051	VIT. B2	Gr	0.02	0.02	0.02
0052	PANTOTH A	Gr	0.03	0.03	0.03
0053	NIACIN B3	Gr	0.04	0.04	0.04
0054	PYRIDOX B6	Gr	0.01	0.01	0.01
0055	FOLIQUE A	Gr	0.00	0.00	0.00
0056	VIT. B12	mg	0.10	0.10	0.10
0057	BIOTINE	mg	0.80	0.76	0.76
0058	CHOLINE	Gr	1.98	2.78	2.78
0058	BiochoLIN	Gr	0.22	0.22	0.22
0061	amidon	%	35.89	27.95	27.95

Copyright A-SYSTEMS
